# Health and working careers of informal carers – what we know and do not yet (but should) know

**DOI:** 10.5271/sjweh.4300

**Published:** 2026-07-01

**Authors:** Jenni Ervasti, Tuija M Mikkola

**Affiliations:** 1 Finnish Institute of Occupational Health, Helsinki, Finland.; 2 Folkhälsan Research Center, Helsinki, Finland.

According to OECD estimates, 13% of adults over 50 years provide – usually unpaid – help with personal care to people with functional limitations, that is, informal care. Of these informal carers, 61% are women caring for a family member (1). As the number of older adults needing care in the EU is projected to rise to 27 million by 2050, the need for informal carers is likely to increase substantially (2). In contrast, the pool of potential caregivers is shrinking due to a smaller number of younger adults, thereby increasing the care burden per working-age person.

Ideally, caring for loved ones is a “win-win-win situation”, where caring brings meaning to the life of the carer, is beneficial for the care recipient, and is highly cost-beneficial to society as it includes far less public spending than formally provided, professional care. However, in many cases, caregiving can be burdensome and lead to long-term stress and health problems. Previous research has linked informal caregiving particularly to mental health risks and work disability due to mental disorders (3–7). Evidence on physical health risks is less clear, with studies indicating higher risks (8, 9), lower risks (10, 11), and no association (7, 12, 13). However, these studies have often been cross-sectional or based only on register data. Therefore, we need stronger evidence with longitudinal designs and more varied outcomes. Moreover, combining the roles of employee and informal carer may lead to additional stress through an imbalance of work and private life, but very little empirical evidence exists thus far. Informal care may also affect work participation, work careers, income, and promotion probabilities, particularly among women (14, 15). More research on informal care, health, and working careers is crucial due to the growing demand for both informal and formal long-term care resulting from population aging.

In addition to more evidence on the health effects of informal care, a major gap in research evidence relates to the role of working conditions in mediating or moderating the association between combining work with informal care and health. There is some evidence that workplace psychosocial factors, such as high worktime control, having a high level of flexibility, good work–private life balance, and social support, may facilitate informal carers’ health and well-being (13, 16–18), whereas job strain in connection with the informal carer role may increase sickness absence (19). To increase knowledge about modifiable factors that have a potential role in shaping the associations between informal caregiving and health, we need more studies on the role of work characteristics, including leadership and other modifiable practices at workplaces, which have thus far received very little attention in longitudinal studies.

A particular risk group with very limited research is individuals with multiple care responsibilities, also known as the 'sandwich generation' carers, who simultaneously provide care for both their aging parents (or in-laws) and their own children. There are also indications that combining highly straining work and informal caregiving is associated with poorer health, particularly among women (19). The reason may be that women more often than men perform high-intensity caregiving, but previous studies have seldom considered caregiving intensity. Aging carers also need more attention as they are the largest age group providing informal care. Thus the role of work characteristics and the health effects of post-retirement informal caregiving need more investigation. Additionally, there is little research on carers who provide both formal care, ie, as healthcare professionals, and informal care. Therefore, more comprehensive studies are needed to understand the long-term health and work disability impacts of caregiving across different population groups, life stages, and work environments.

Yet another research gap lies in the lack of international comparisons. Countries differ based on how care is organized; who is supposed to provide informal care; who is supposed to work; and until what age people (men and women) are supposed to work. The structural frameworks defining how care is provided, organized, and divided between the state, market, and family within a country are called care regimes (20). It may be that the health and subsequent work disability effects of informal care differ between different care regimes. In the European Nordic countries, the care regime has traditionally been characterized by a high level of state intervention, public spending, and reliance on professional care. In Central Europe, care is traditionally provided by a mix of public and private providers, voluntary or religious non-profit organizations, and moderate levels of family responsibility. In the Mediterranean/Southern Europe and in the majority of Eastern Europe, traditional care regimes rely heavily on families, particularly women, who are expected to provide informal care (20). The labor markets are organized according to these traditional care regimes as the regime is reflected, for example, in gender- or non-neutral retirement age and expectations of both sexes to equally or unequally participate in the labor market. However, due to political changes, economic downturns, and cuts to public spending, there is increasing pressure for informal care even in countries where the traditional care regime has emphasized high reliance on professional care and all people of working age are expected to participate in the labor force (21, 22). This may create a double or triple burden of caring for one’s own family, disabled or aging relatives, and simultaneously holding full-time paid employment.

We need more research to inform policymakers across Europe as increasing informal care may conflict with the goal of prolonged working lives and healthy aging through a higher age of old age pension. If informal carers are burdened to the extent that they themselves need formal healthcare, the goal of decreasing the costs of formal care cannot be met. Flexibility in terms of worktime control has been linked, in addition to the health of informal caregivers, also to extending work careers beyond retirement age (23). Governments, decision-makers, workplaces, and occupational health care need information on these modifiable factors to develop policies to support sustainable working lives for people with different care responsibilities in private life.
